# Gentian Root as a Dilator

**Published:** 1868-01

**Authors:** 


### Gentian Root as a Dilator.

Professor Winckel in Rostock, recommends (Deutsche Klinik, 1867) the radix gentianæ rubræ as a new, simple, and cheap means of dilatation for surgical and gynecological purposes. His attention was first directed thereto by an article of John Jacob Haeberl, published in 1834, in which the author states that having operated for atresia uteri and desiring to keep open the orifice made by the trocar, he introduced a good, firm plug of radix gentianæ, and that on the following day he found no small difficulty in withdrawing the same, which had increased to twice its former size. According to Dr. Winckel's observations the gentian root has the following advantages over laminaria: 1st. Its cheapness, the ease with which it can be obtained, and the fact that the physician can so easily cut plugs and bougies of any size to suit his requirements. 2d. Its somewhat smaller power of absorption, as compared with laminaria, is compensated by our being able to obtain larger pieces of it (one and one-half to two inches in diameter) so that it can be used for the dilatation of openings already too large for laminaria. 3d. The fact of its *remaining free from smell* constitutes an immense advantage, for even laminaria, though in a much less degree than sponge-tents, often becomes quite fœtid.

The radix gentianæ may therefore be used with special advantage in strictures of the vulva, vagina, and uterus; for tamponing the uterus in smaller hæmorrhages, for the induction of abortion, for dilatation after operations for atresia of the genital organs. Whether it is also applicable to stricture of the urethra, to affections of the lachrymal ducts, etc., remains to be seen.—*All. Med. C. Zeitung*, 1867.

